# A simple goiter of the right ventricle: case report and literature review

**DOI:** 10.3389/fcvm.2024.1497659

**Published:** 2024-11-18

**Authors:** Shuai Luo, Xiaoxue Tian, Ting Xu, Jinjing Wang

**Affiliations:** Department of Pathology, Affiliated Hospital of Zunyi Medical University, Zunyi, Guizhou, China

**Keywords:** right ventricle, teratoma, pathology, goiter, treatment

## Abstract

**Background:**

Cardiac teratoma is extremely rare, accounting for less than 1% of adult cardiac tumors. These teratomas typically occur in the pericardium and myocardium, with intracardiac teratomas being even rarer. Given the limited number of cases, diagnosing and treating intracardiac teratomas remains challenging.

**Case demonstration:**

A 50-year-old man was admitted to the hospital with a 1-month history of chest tightness and shortness of breath after exertion. Color Doppler echocardiography revealed a hyperechoic mass of approximately 58 mm in diameter in the right ventricular cavity and outflow tract. Postoperative pathological examination confirmed a right ventricular monodermal teratoma (goiter). The patient was followed up for 2 years with good overall health and no recurrence.

**Conclusions:**

Intracardiac teratomas are exceedingly uncommon tumors, with the predominant right ventricle involvement being observed across a wide age range. These teratomas are often histologically classified as benign. The early detection of intracardiac teratomas relies on imaging findings, while a definitive diagnosis requires histopathological examination. The primary treatment is surgical resection, which yields a favorable prognosis.

## Background

Cardiac teratoma is an extremely rare condition that represents approximately less than 1% of adult cardiac tumors (1), with only case reports documented in the literature. The clinical manifestations of these teratomas primarily depend on the anatomical location of the tumor, while they can be histopathologically classified as benign or malignant. Cardiac teratomas often arise in the pericardium and myocardium. However, intracardiac teratomas are even rarer, with no more than 10 cases reported to date. According to the World Health Organization definition (2), mature cystic teratomas (MCTs) are composed entirely of mature tissue derived from two or three germ layers (including the ectoderm, mesoderm, and/or endoderm). Conversely, monodermal teratomas, which arise from a single germ layer, are rarer and include goiters, carcinoids, and neuroectodermal tumors. Cardiac teratomas are mainly MCTs (3), with no published cases of intracardiac monodermal teratomas. This case report presents the first documented case of a cardiac monodermal teratoma (goiter), aiming to improve the current understanding of this rarely occurring tumor.

## Case demonstration

A 50-year-old male patient was admitted to the hospital with a 1-month history of chest tightness and shortness of breath after exertion, without amaurosis fugax, incontinence, cough, expectoration, cold, and fever. Physical examination revealed an enlarged left heart border, a heart rate of 80 beats/min, a regular heart rhythm, and a rumbling murmur in the apex of the heart.

**Figure 1:**
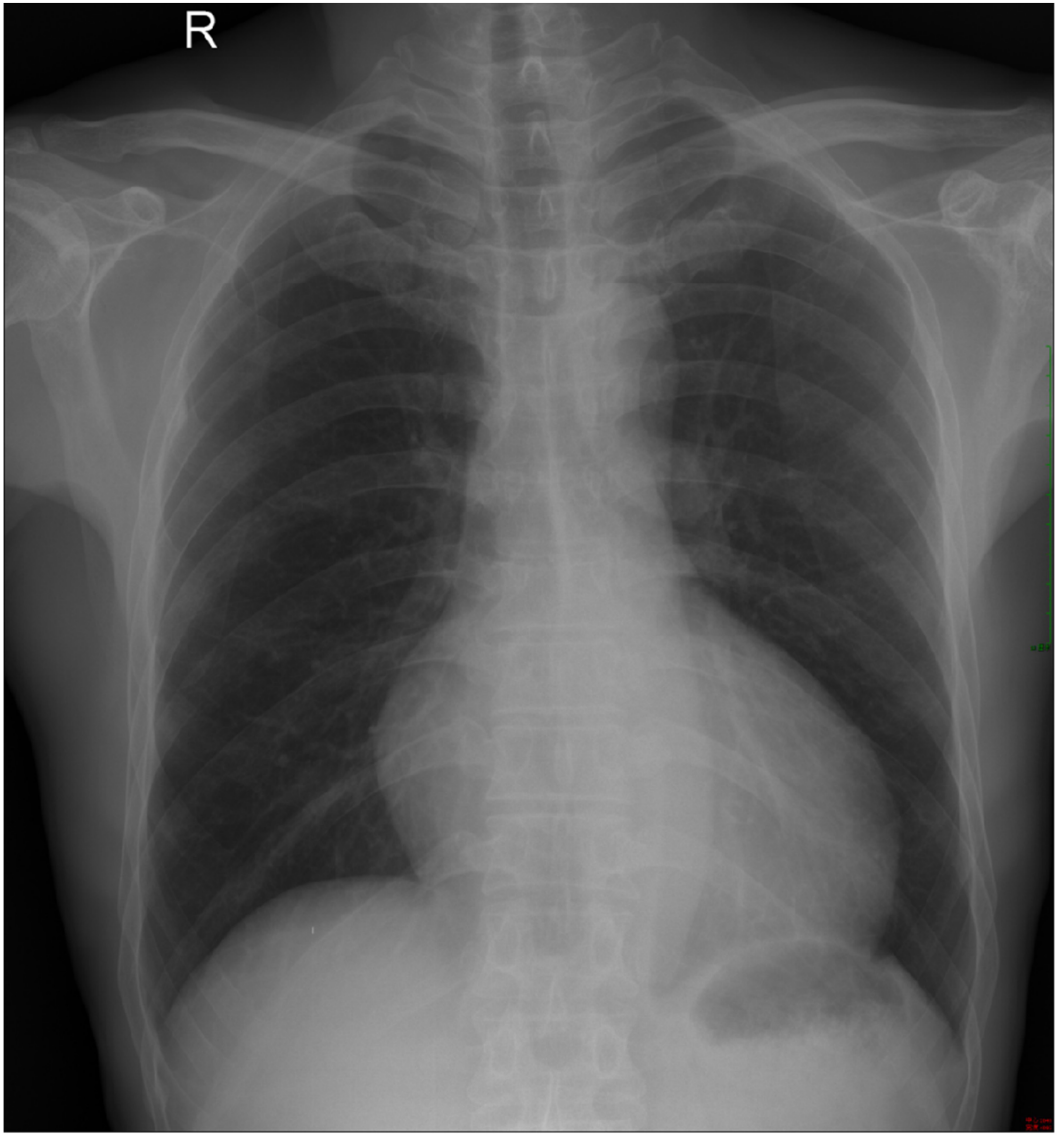
Chest radiograph showed an increased transverse diameter of the heart shadow, a smooth bilateral diaphragmatic surface, and a sharp costophrenic angle.

**Figure 2:**
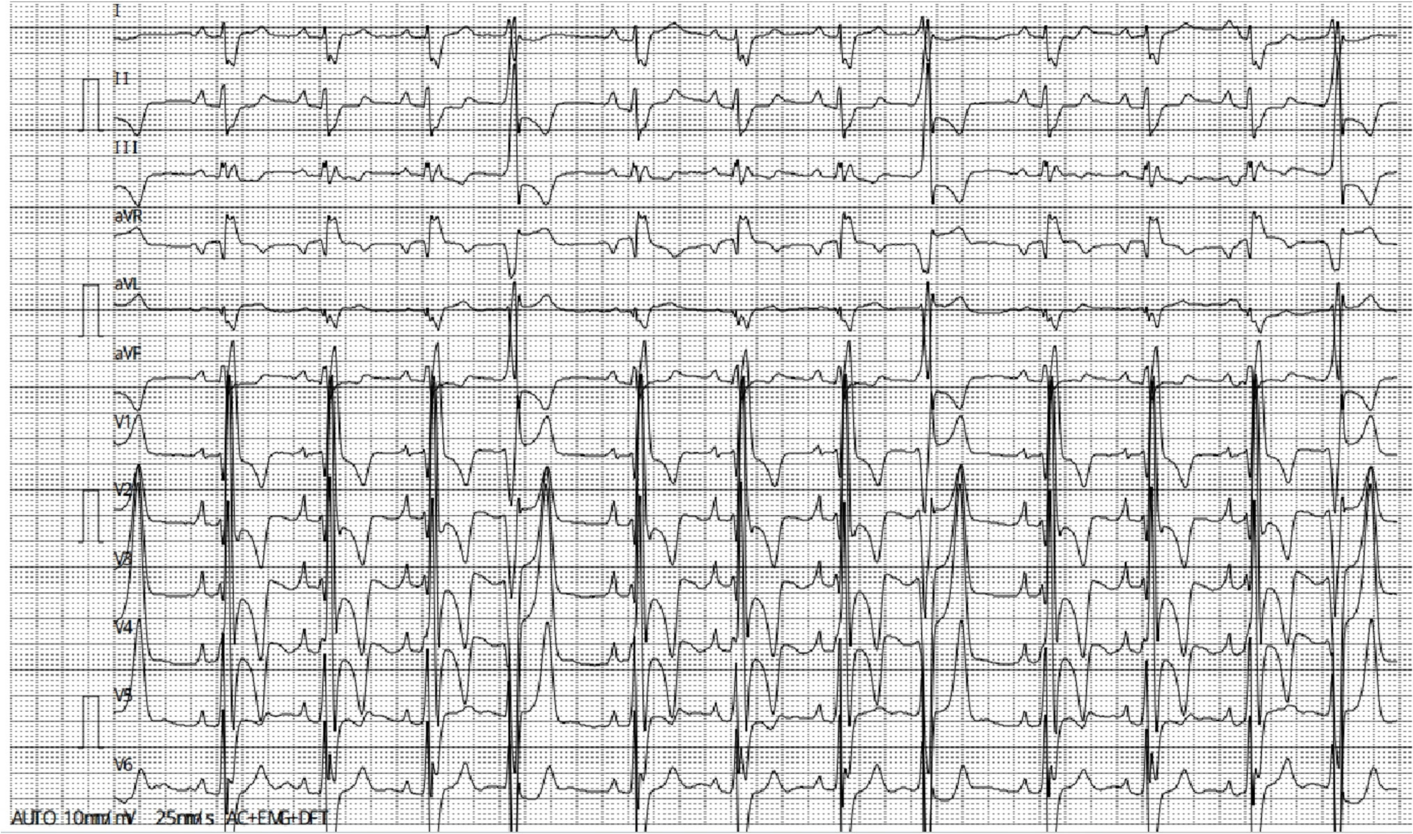
Electrocardiography demonstrated right atrial hypertrophy, complete right bundle branch block, and premature ventricular contraction.

**Figure 3:**
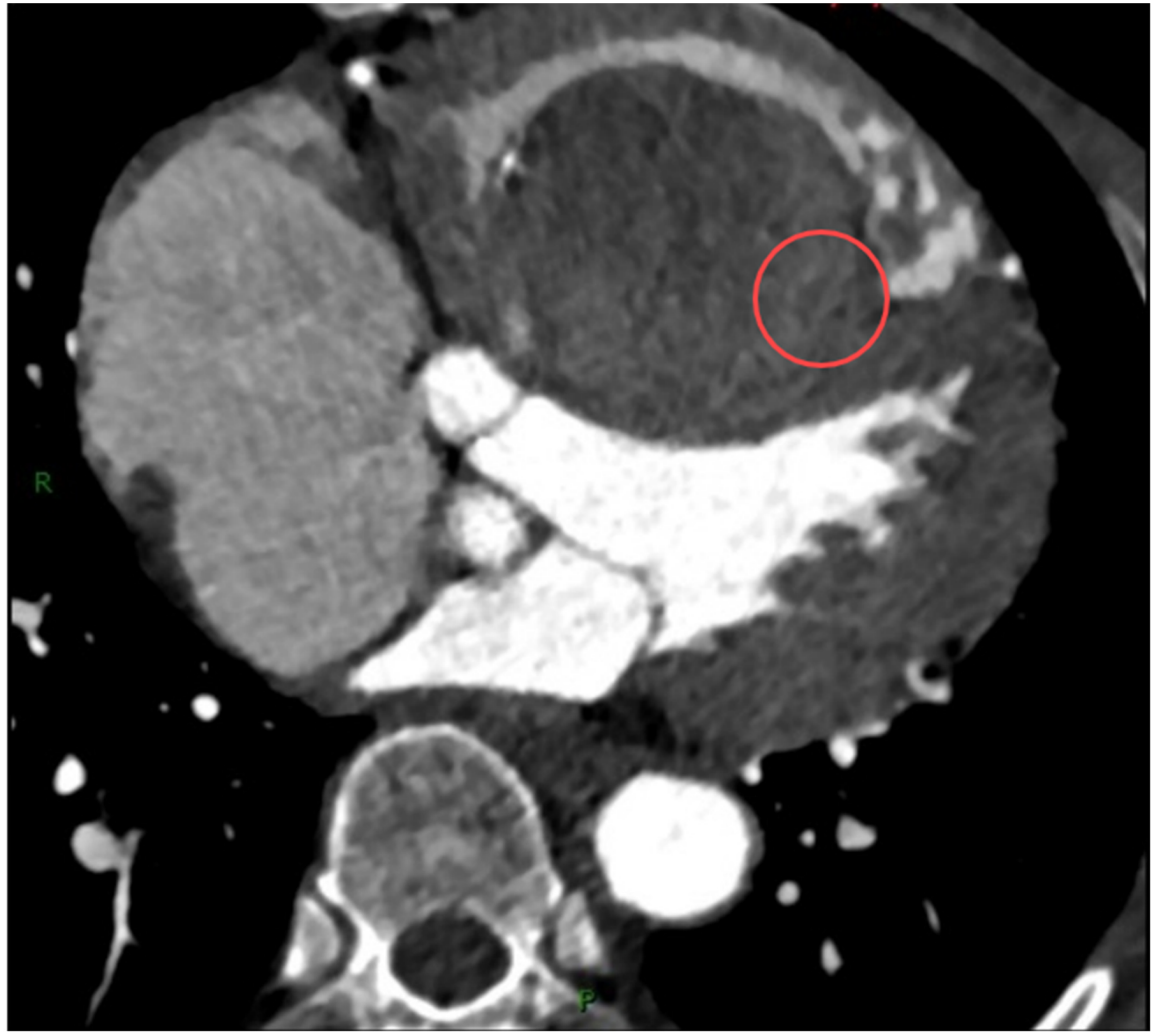
Contrast-enhanced CT scan showed a massive filling defect in the right ventricle with uneven enhancement (marked with red circles).

**Figure 4:**
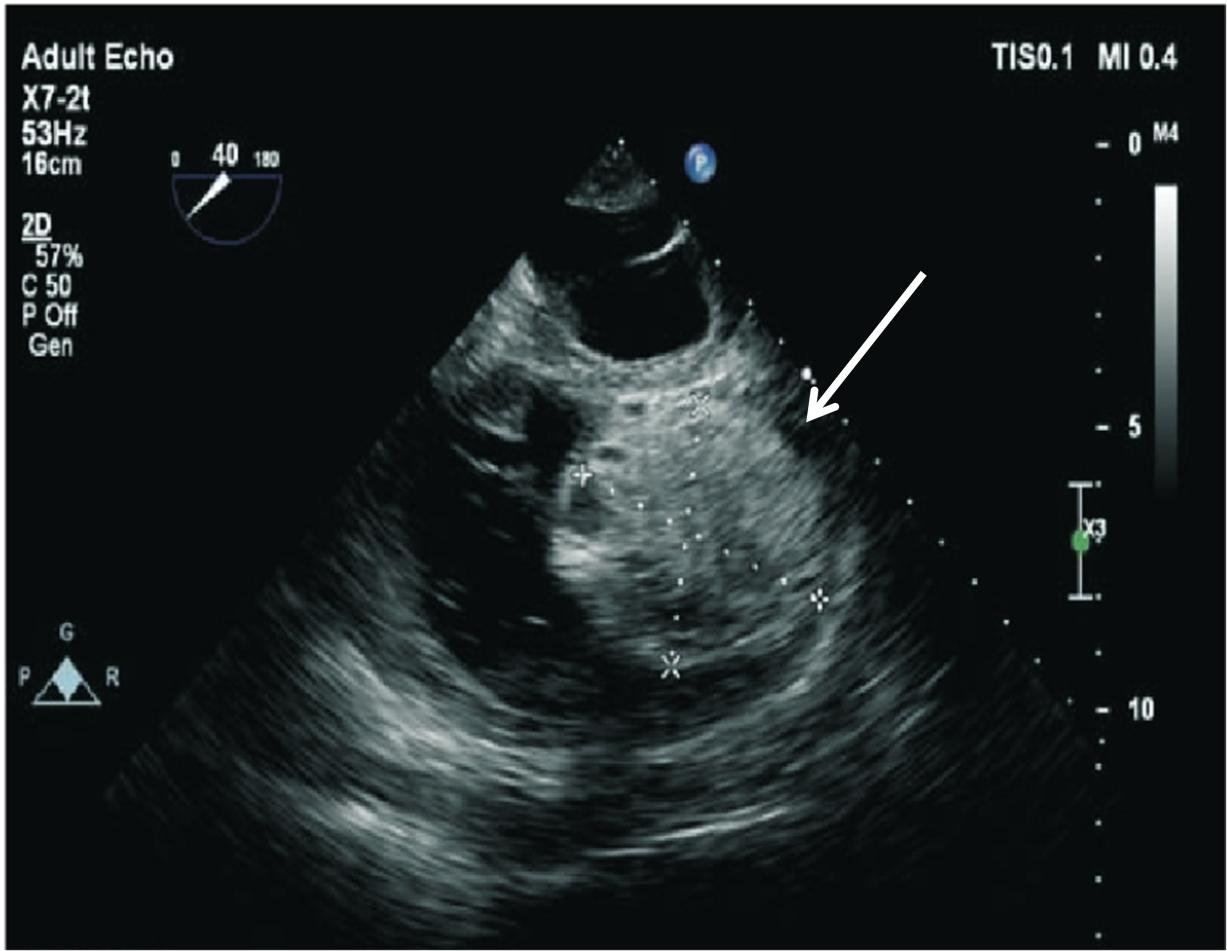
An echocardiogram revealing a hyperechoic mass in the right ventricular cavity and outflow tract (white arrow).

A chest radiograph (Figure 1) showed an increased transverse diameter of the heart shadow, a smooth bilateral diaphragmatic surface, and a sharp costophrenic angle. Electrocardiography (Figure 2) demonstrated right atrial hypertrophy, complete right bundle branch block, and premature ventricular contraction. Chest computed tomography (CT) (Figure 3) and coronary angiography further demonstrated enlargement of the heart, obvious right atrium and ventricle enlargement, and a massive filling defect of 51 mm × 60 mm in the right ventricle accompanied by uneven density and punctate calcification, which showed enhancement on the contrast-enhanced scan. The left and right coronary arteries arise from the left and right coronary sinus, respectively, and are right-sided dominant. Non-calcified plaque was seen in the distal segment of the right coronary artery, corresponding to mild-to-moderate luminal stenosis. Calcified plaque was found in the proximal segments of the left anterior descending and middle coronary arteries, which corresponded to mild luminal stenosis. Echocardiography indicated that the right atrium and right ventricle were significantly enlarged, while a hyperechoic mass approximately 58 mm × 48 mm in size was observed in the right ventricular cavity and outflow tract (Figure 4). The base of this mass was attached to the basal segment of the anterior septum and showed poor mobility. Furthermore, the inner diameter of the aortic sinus was widened, whereas the inner diameter of the pulmonary artery was normal. The thickness of the interventricular septum and the left and right ventricular walls was normal, while the left ventricular posterior wall exhibited slightly reduced motion. No obvious abnormalities were found in the morphology and structure of the valves. In addition, a Doppler examination revealed moderate eccentric tricuspid regurgitation and mild aortic valve regurgitation. The pulmonary artery systolic pressure was estimated to be approximately 53 mmHg, owing to the tricuspid regurgitation. Mitral flow spectrum showed that peak E was greater than peak A. Based on these findings, the radiologist considered the possibility of thrombosis.

In light of the patient's clinical manifestations of a right ventricular mass, severe tricuspid regurgitation, and cardiac insufficiency, enucleation of the right ventricular tumor and tricuspid valvuloplasty were performed. In this procedure, the right atrium and atrial septum were explored, and a mass of approximately 6 cm × 5 cm was found in the anterior septum of the right ventricle, along with a relatively smooth, pedunculated capsule and a slightly wide base measuring approximately 2.5 cm × 1.5 cm.

Pathological examination revealed a gray-brown oval tissue (6.5 cm × 4.5 cm × 2.5 cm) in size, with a complete capsule, clear boundary, gray-red cut surface, soft texture, local jelly-like, hemorrhage, and calcification.

**Figure 5:**
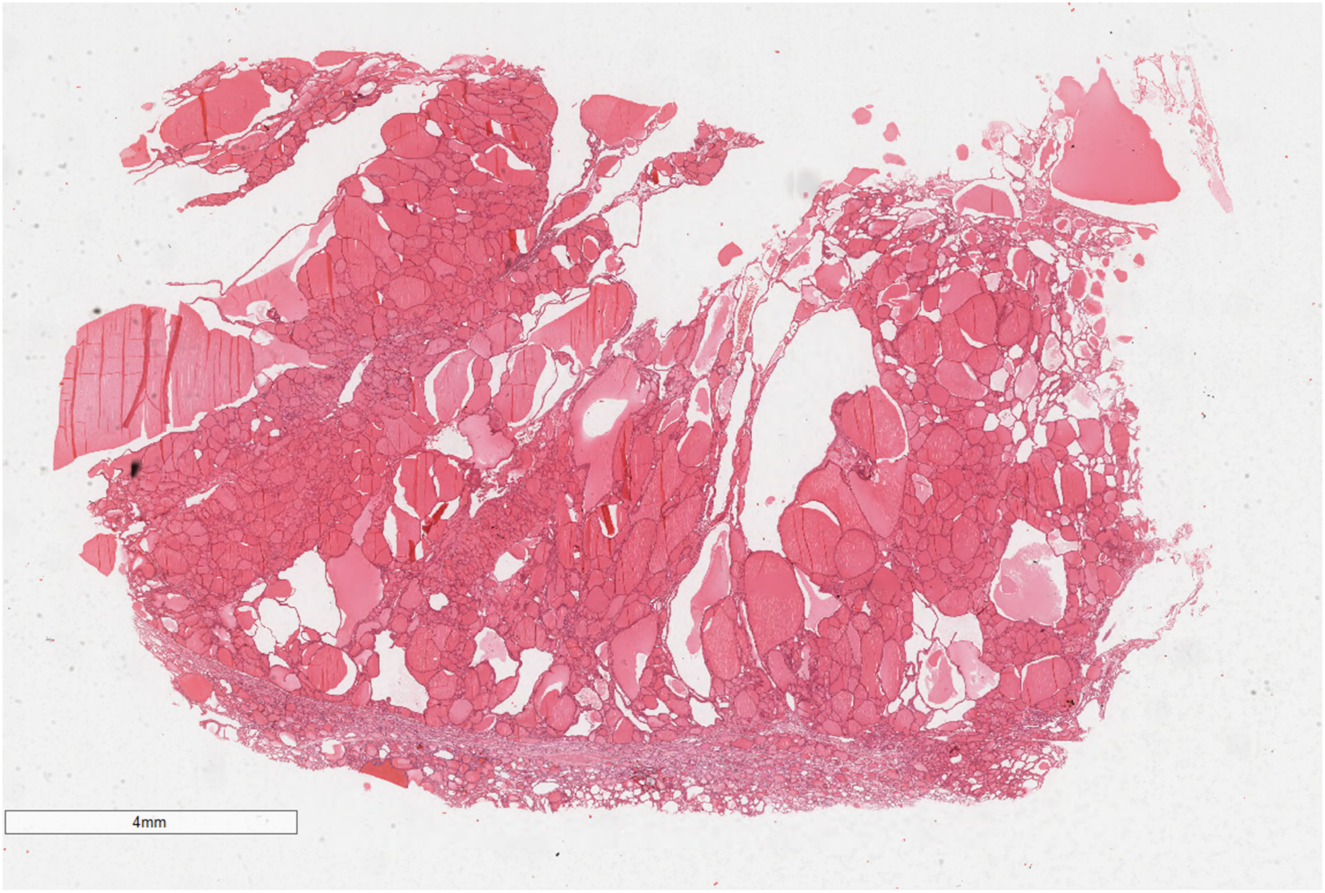
The mass was well-circumscribed and consisted almost entirely of normal or hyperplastic thyroid tissue. Hematoxylin and eosin, ×7.

**Figure 6:**
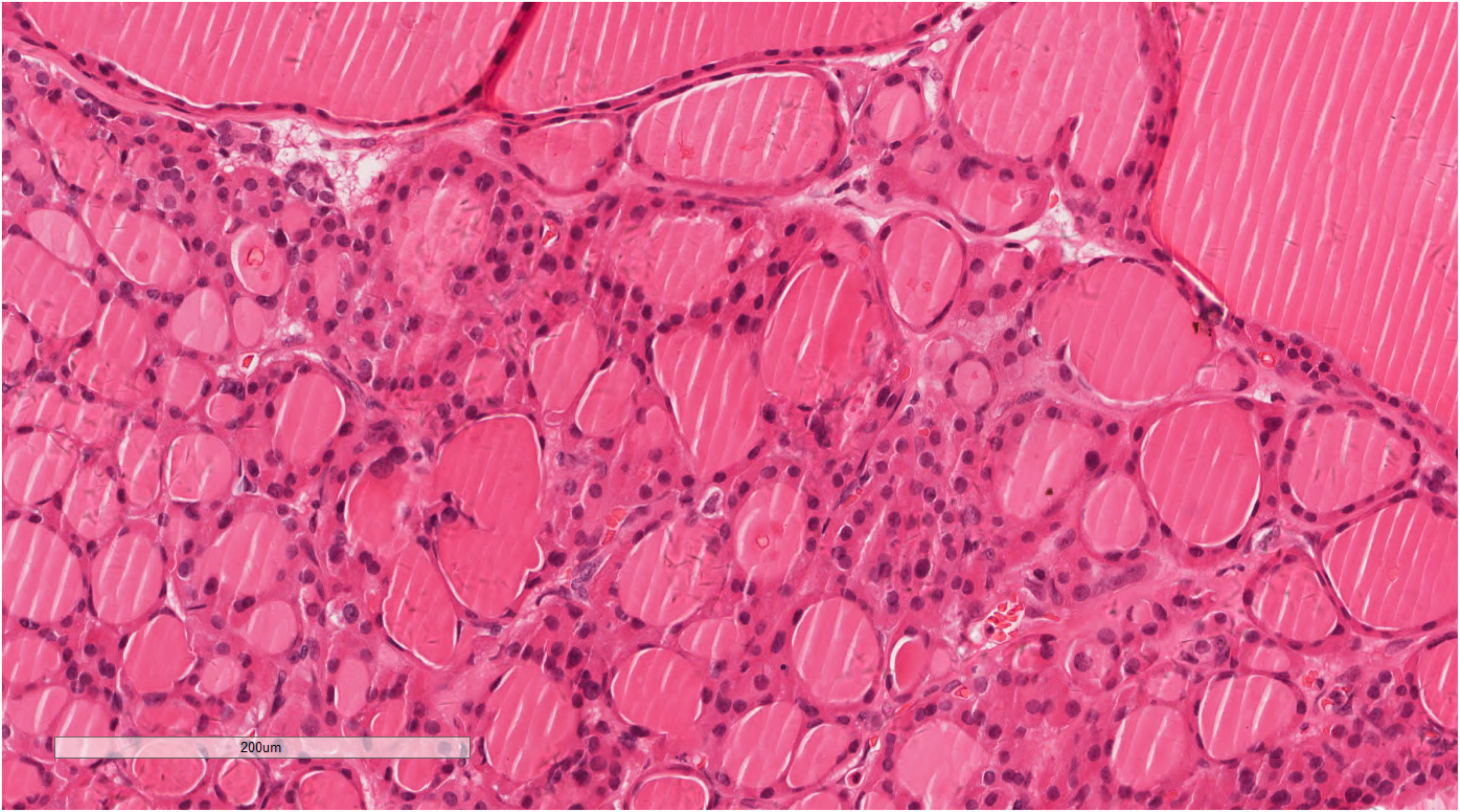
A high magnification view demonstrating a single layer of low columnar or cuboidal follicular epithelium, with mild morphology. Hematoxylin and eosin, ×200.

**Figure 7:**
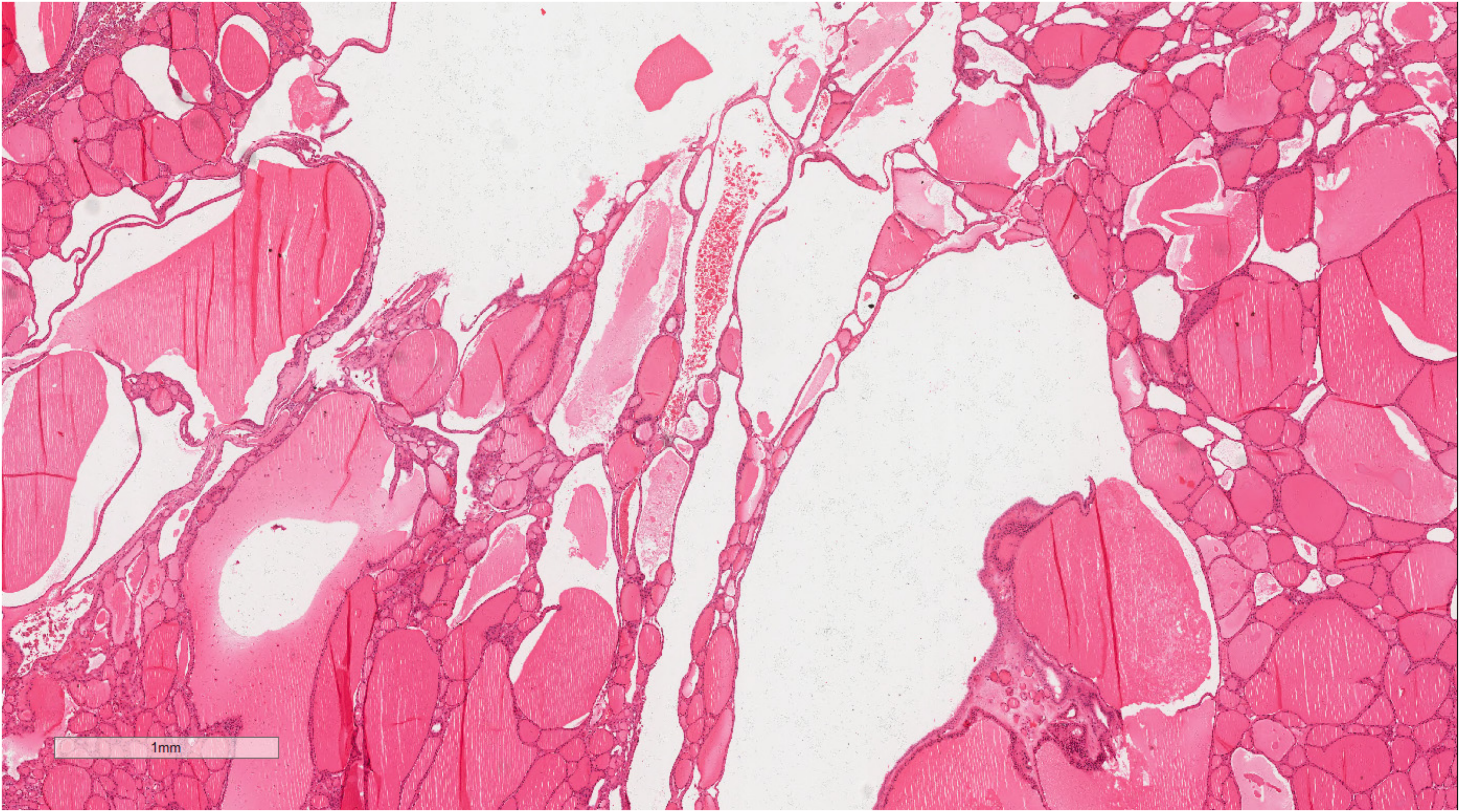
The tumor showed local hemorrhage and cystic degeneration. Hematoxylin and eosin, ×22.

Histological evaluation showed that the lesions comprised normal or hyperplastic thyroid tissue of varying sizes and mature thyroid follicles (Figure 5). The size of the follicles ranged from microfollicles to giant follicles, and the cavity consisted of eosinophilic glial material. A high magnification view (Figure 6) demonstrated a single layer of low columnar or cuboidal follicular epithelium, bland, with a small proportion of eosinophilic, clear, and mucous cells. Local hemorrhage and cystic degeneration were also visible (Figure 7).

Based on the histopathological features, the tumor, which was composed entirely of thyroid tissue without other teratoma components, was diagnosed as a right ventricular monodermal teratoma (goiter).

After surgical resection of the ventricular mass, the patient was followed up for 2 years, during which he exhibited good overall health and no recurrence.

## Discussion

Primary cardiac teratomas are excessively rare, typically developing in the pericardium and myocardium, with intracardiac teratomas being even more uncommon. Currently, only four case reports of teratomas in the ventricular cavity have been published (3–6). Consequently, those previous cases along with the present one lead to five cases (Table 1).

**Table 1:** Clinicopathological features of intracardiac teratomas and literature review.

	Time		Sex	Age (years)	Diameter (cm)	Location	Symptoms	Primary (metastasis)	Histologic types	Treatment and prognosis
1	1998	Campagne et al. (3)	F	29	4	Ventricular septum, right ventricle	Syncope	Primary	Mature cystic teratoma	Found at autopsy
2	2019	Farid et al. (5)	F	9 days after birth	1.5	Right ventricle	Recurrent syncope	Primary	Mature cystic teratoma	Recovered well after surgical resection
3	2021	Abdulla et al. (4)	M	22	11.7	Right ventricle	Progressive dyspnea, palpitations, and syncope	Testicular teratoma metastasis	Malignant teratoma of germ cell	Complete regression was achieved after chemotherapy
4	2021	Tao et al. (6)	M	37	1.2	Right ventricle	Chronic chest pain and palpitations worsened	Primary	Mature cystic teratoma	Recovered well after surgical resection
5	2024	This case	M	50	5.8	Right ventricle	After fatigue, chest tightness, shortness of breath	Primary	Monodermal teratoma (goiter)	Recovered well after surgical resection

Among these five cases of intracardiac teratoma, three were diagnosed in male patients and two in female patients, with a mean age of 28 years. The tumors had a mean diameter of 4.8 cm, with all cases involving the right ventricle. In terms of clinical manifestations, three patients had syncope, while two presented with palpitation, dyspnea, and chest pain and tightness. Among these five cases, four were primary cardiac teratomas and one was a testicular teratoma metastasized to the heart. Of the four cases of primary cardiac teratomas, three were MCTs and the fourth is the first case of cardiac monodermal teratoma (goiter) documented in the current report. In terms of treatment outcome, three cases of primary cardiac benign teratomas showed good results after surgical treatment. In the case of the testicular malignant teratoma metastasized to the heart, complete tumor regression was achieved after chemotherapy. The fifth remaining case was diagnosed at autopsy.

Intracardiac teratomas generally occur over a broad age range, with most cases predominantly developing in the right ventricle of young and middle-aged patients. Furthermore, MCT is the most prevalent histological type. The clinical manifestations of these teratomas are primarily determined by the anatomical location of the tumor rather than its histopathology. The rapid growth of cardiac teratomas may be attributed to the rich blood supply in this region, which provides a conducive environment for tumor growth and increases the likelihood of serious mechanical consequences due to excessive volume. Moreover, different pathogenesis can lead to complications, such as embolism and heart valve impairment, which in turn affect the blood supply function of the heart to cause symptoms of systemic ischemia and hypoxia (7), including syncope, dyspnea, and palpitation. Given that MCTs are the most frequent histological type, this case report is of notable significance as the first documented description of a patient with a cardiac monodermal teratoma (goiter). The most commonly used and valuable methods for the early detection of cardiac-occupying lesions are non-invasive cardiac color Doppler ultrasound and CT, with cardiovascular magnetic resonance (CMR) also emerging as a crucial tool to evaluate cardiac malignant tumors (8). Surgical treatment is the preferred strategy in symptomatic patients, whereas a close follow-up is favored in asymptomatic patients (9).

## Conclusion

Intracardiac teratomas are extremely rare tumors that predominantly occur in the right ventricle across a wide age range. These teratomas are usually histologically classified as benign, with clinical manifestations such as ischemia and hypoxia primarily determined according to the anatomical location of the tumor and the degree of obstruction. Symptomatic patients generally undergo surgical treatment, while asymptomatic patients are managed with close follow-up.

## Data Availability

The original contributions presented in the study are included in the article/Supplementary Material, further inquiries can be directed to the corresponding author.
